# SW#db: GPU-Accelerated Exact Sequence Similarity Database Search

**DOI:** 10.1371/journal.pone.0145857

**Published:** 2015-12-31

**Authors:** Matija Korpar, Martin Šošić, Dino Blažeka, Mile Šikić

**Affiliations:** 1 University of Zagreb, Faculty of Electrical Engineering and Computing, Unska 3, HR 10000 Zagreb, Croatia; 2 Bioinformatics Institute, A*STAR, 30 Biopolis Street, #07–01 Matrix, 138671 Singapore, Singapore; Aberystwyth University, UNITED KINGDOM

## Abstract

In recent years we have witnessed a growth in sequencing yield, the number of samples sequenced, and as a result–the growth of publicly maintained sequence databases. The increase of data present all around has put high requirements on protein similarity search algorithms with two ever-opposite goals: how to keep the running times acceptable while maintaining a high-enough level of sensitivity. The most time consuming step of similarity search are the local alignments between query and database sequences. This step is usually performed using exact local alignment algorithms such as Smith-Waterman. Due to its quadratic time complexity, alignments of a query to the whole database are usually too slow. Therefore, the majority of the protein similarity search methods prior to doing the exact local alignment apply heuristics to reduce the number of possible candidate sequences in the database. However, there is still a need for the alignment of a query sequence to a reduced database. In this paper we present the SW#db tool and a library for fast exact similarity search. Although its running times, as a standalone tool, are comparable to the running times of BLAST, it is primarily intended to be used for exact local alignment phase in which the database of sequences has already been reduced. It uses both GPU and CPU parallelization and was 4–5 times faster than SSEARCH, 6–25 times faster than CUDASW++ and more than 20 times faster than SSW at the time of writing, using multiple queries on Swiss-prot and Uniref90 databases

## Introduction

Searching for protein homologues has become a daily routine for many biologists. Popular BLAST tools (PSI/DELTA/BLASTP) [1–3] produce search results for a single query in less than a second and many bioinformatical tools have come to depend upon the BLAST tool family to find matches in the database of sequences. However, protein sequence databases are growing at an unprecedented pace and we would often like to find homologous of not one, but hundreds, thousands, or more queries. When using existing tools, the extensive time cost of such a search can hinder the research. BLAST family of tools, not being naturally parallelisable, is unable to utilize the development of new hardware focused on low level parallelism (inter-core and many-core architectures). What characterizes dynamic programming algorithms such as Smith-Waterman [4] is that they provide maximum sensitivity at the cost of long running times (due to their quadratic time complexity). The initial heuristic tools were developed for this purpose precisely–to sacrifice some sensitivity, and achieve shorter running times.

One major feature of these initial heuristic tools, such as BLAST, is that they perform the alignments not on the original, but on a reduced database which gets heuristically determined in the first step of every search. Sizes of queries and databases today have rendered even such an optimization insufficient, with the main bottleneck usually being the second, alignment step. For this reason we propose SW#DB–a standalone tool and a library that perform efficient exact alignment step using dynamic programming algorithms on a reduced database utilizing both NVIDIA CUDA (Compute Unified Device Architecture) and CPU SIMD (Single Instruction Multiple Data) instructions. Although the Sw#db tool enables the use of global Needleman-Wunsch algorithm and semi-global algorithm, its focus is on local the Smith-Waterman algorithm. In comparison with other parallelized implementations of the Smith-Waterman algorithms for sequence similarity search, this implementation is additionally optimised for multiple queries rendering it significantly faster than both the state-of-the-art CUDASW++ [5–7] GPU enabled database search tool and SIMD-optimized tools such as SSEARCH [8] and SSW [9]. It also supports multiple GPU cards and could be run on clusters. Although GPU implementations of the BLAST algorithm exist (i.e. GPU-BLAST and CUDA-BLASTP) it was shown that they perform worse than original NCBI BLAST on multi-core architectures [10]. Therefore we do not include them in the comparison.

Although the running times of SW#db for searching the whole database are comparable with those of the BLAST family of tools, our main intention here is to provide an open source library for the alignment step of the database search tools. Our motivation was to give the researchers control over which and how many of target sequences interest them. For this reason we provide functionality of choosing a subset of seed sequences from which to generate exact alignments—enabling researchers to experiment and tailor the results to their need. SW#db is optimised for simultaneous computation of the alignments and due to its architecture; it is much faster than the implementations of alignment phases used in BLAST tools. The library could be used not only for protein database search based on the local protein alignment, but it could be also be used for the global or semi-global alignment of both protein and nucleotide queries on databases. Due to quadratic complexity of the dynamic programming algorithms, we do not recommend it being used for searching nucleotide databases with long sequences.

## Methods

The Smith-Waterman algorithm provides the alignment score and the optimal alignment path for a pair of sequences, with quadratic complexity both in time and memory. The implementation of the Smith-Waterman algorithm for sequence similarity search can divided into two phases: (i) Scoring phase which provides the alignment score with quadratic time and linear memory complexity and (ii) Reconstruction phase which provides the alignment paths for n best scored pairs. In practise *n* is significantly smaller than the number of sequences in the database which has focused most of the optimization efforts on phase (i) Optimizations of the scoring phase rely on hardware architecture that provides a way to score multiple pairs of sequences in parallel. Two of the most popular hardware architectures that support parallelization are CPUs and GPUs. Most modern CPU designs enables SIMD (single instruction multiple data) instructions [11]. SIMD allows parallel execution of a single instruction on multiple data. Amount of data that can be processed at a time depends on the size of the data and the SIMD version. The most dominant architecture and the framework to program GPUs is NVIDIA CUDA. CUDA GPU architecture allows massive multithreading and has been used to optimize the Smith-Waterman scoring phase. CUDA cards with CUDA architecture 3.5 or higher support SIMD usage on GPUs. In SW#, both of the afore mentioned architectures are utilized if available on the system.

SW#db uses OPAL library [12] to utilize CPU SIMD architectures. OPAL enables fast sequence similarity search using the Smith-Waterman algorithm. It works the most efficient when it uses 8 bit arithmetic. Since the width of SIMD register file is up to 128 bits Opal can calculate up to sixteen sequence alignments at a time. Since the signed arithmetic is used to prevent overflow, the maximum possible score is 127. For overflow detection intrinsic SIMD functions are used. They check whether the result is equal to 127. If the score reaches this maximum value it is recalculated in 16 bits. Very rarely it is necessary to use 32 bit (four bytes) arithmetic. In the cases when the recalculation is necessary, the SIMD parallelization is not used what slowing down execution.

Execution time speedups offered by CUDA architecture depend on various factors, such as branch divergence: if every CUDA thread runs exactly the same code, the speedup will be maximized. In the case of the Smith-Waterman algorithm branch divergence can be avoided only if all of sequences in the database are the same length. With the increasing difference in database sequence length, the CUDA parallelization overhead increases. Therefore, local alignment calculation done by CUDA is divided into two parts, the part that handles database sequences of similar length- called the short kernel, and the part that handles sequences with a large difference in length—the long kernel. Because the length difference is lower between shorter sequences, sequences in a database are divided into short and long sequences by the predefined length threshold, *L*. A disadvantage of this method is that it depends on the distribution of sequence lengths in the input database. Usually most of the input sequences are shorter than the predefined length threshold. The default threshold value is 2000.

**Fig 1:**
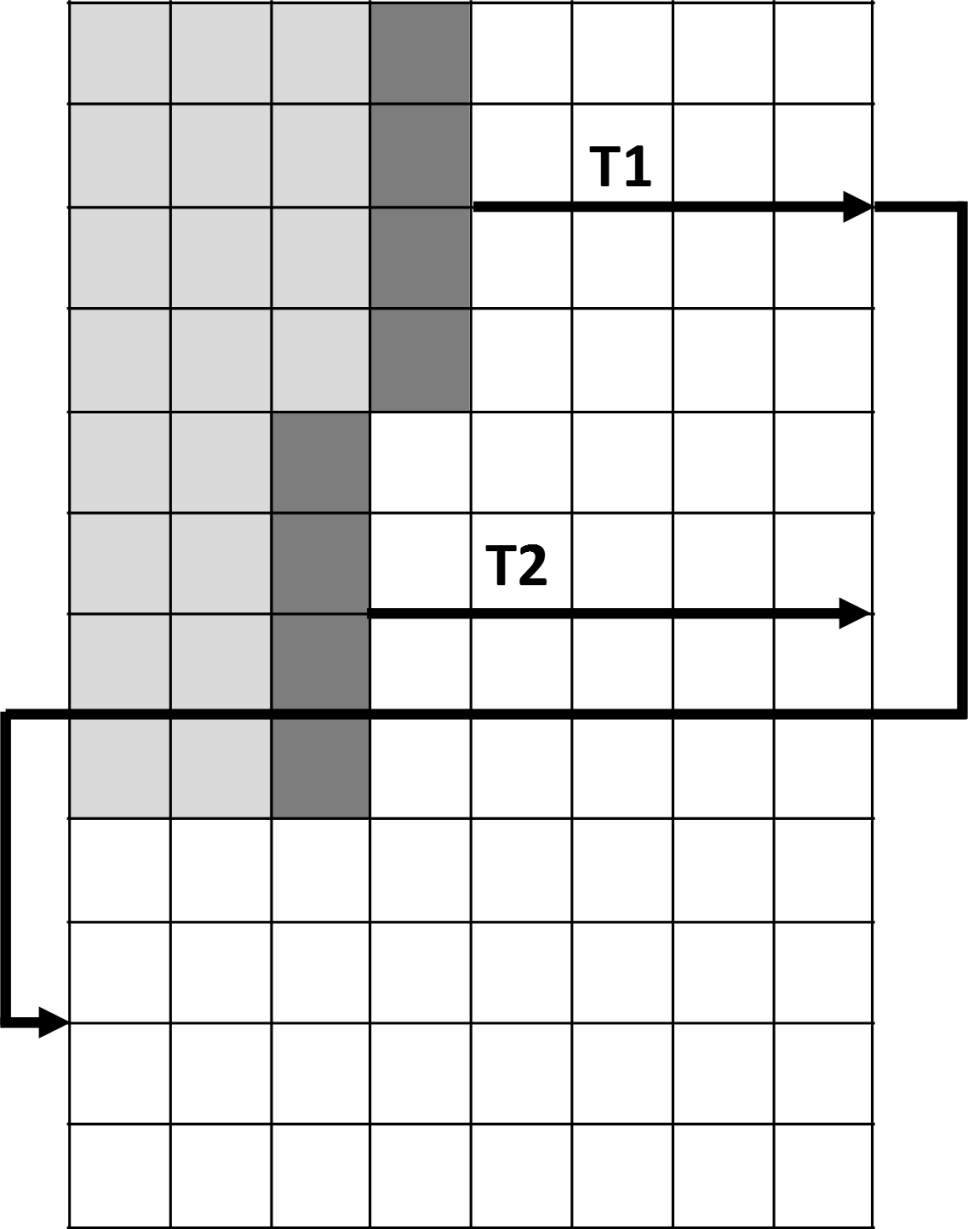
GPU long kernel execution. Each thread in SW#db long kernel solves four rows using optimized CUDA structures.

The number of CUDA blocks and threads will be denoted as *B* and *T*, respectively. Short kernel scores *B* × *T* sequence pairs at a time, each CUDA thread scores single input query sequence with a database sequence. Memory complexity of the short kernel is 2 × *n* × *m*, where *n* is the number of short sequences in the database, and *m* is the length of the longest short sequence. Long kernel scores *B* sequences at a time, each CUDA block scores a single pair of sequences with *T* threads. Long kernel has memory complexity of 9 × *n*, where *n* is the sum of lengths of long database sequences. The method used in long kernel is similar to the method used in [13] for the pairwise Smith-Waterman alignment parallelization. Each thread in the block solves four rows at a time in an antidiagonal, wavefront manner (Fig 1). When thread *Ti* solves a row, and there are still unsolved rows left, it starts to solve row *i* + *T* * 4, where *T* is the number of threads in the block. Conversely, in [5] every thread solves $\frac{n}{T}$ rows, where *n* is the sequence length. SW#DB uses optimized CUDA four vector elements which require a lower number of CUDA registers and therefore lower execution time.

**Fig 2:**
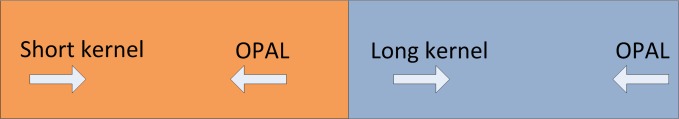
Database processing steps. Sequences in the database are sorted by their length and divided into two partitions. In the both partitions GPU kernels (short and long) process from shorter to longer sequences and OPAL (CPU implementation) processes in the opposite direction.

Since the efficiency of both GPU and CPU algorithm is heavily dependent on sequence lengths, we applied a scalable alignment method (Fig 2). In the preprocessing step the sequences in the database are sorted by their length. Short kernel starts by scoring the shortest sequences. CPU part (OPAL) starts by scoring the longest sequences. Additionally, another instance of OPAL starts scoring sequences shorter than *L* Short kernel stops when it reaches sequences already scored by the second instance of OPAL. The first OPAL instance stops when it scores all sequences longer than *L*, or it is stopped when short kernel ends. If short kernel ends before the first OPAL instance, all sequences longer than *L* and not solved by the first OPAL instance are solved by the long kernel. This approach scales well with different CPU speeds, the number of CPU cores and the performances of available GPUs. This kind of dynamic work delegation, which does not depend on any predefined parameters, minimizes thread waiting times and significantly reduces the execution time. This method scales very well on multi-query database alignments. The pseudo code and detailed flowchart of this method are presented in **S1 Algorithm** and **S1 Fig**, respectively.

When CUDA cards with CUDA architecture 3.5 are available, the previously explained scoring process is run twice. In the first run, scores are limited to 127, single byte arithmetic is forced, to maximize the SIMD speedup for both CPU and GPU SIMD architectures. In the second run SIMD is disabled and only sequences with the score equal to 127 are recalculated. Very often it is necessary to score a subset of database sequences. This approach can be used for sequence similarity database search when in the first step we reduce the number of candidate sequences. In that case we would need to prepare a new database for every query sequence and load it in the GPU memory which would significantly increase execution time. To allow scoring of only a subset of the database sequences, without preparing a new database, we propose the usage of database indexes. Indexes are ordinal numbers of sequences in the database. For a given input array of database sequences and the whole database prepared in advanced, SW#DB scores only the sequences with given indexes. In the short kernel the work delegation needs to be reconfigured to minimize the overhead produced by the difference in indexed sequence lengths. This is done by masking the thread to data fetching method. When loading data via textures, not only is the requested data loaded in the working memory, but also the data from the neighbouring memory address is loaded into cache. Therefore, the only overhead is in the global memory texture cache misses, because of the potential memory address distance between two neighbor indexed sequences. In this manner, depending on the number and nature of indexed sequences, indexed solving is at least as fast as the solving of the whole database. As the number of indexed sequences lowers, so does the execution time. This method is used for score recalculation in the second pass of CUDA SIMD scoring method.

SW#db is available as a library through a C API, as well as a standalone executable command-line tool. A standalone executable, which utilizes MPI for database alignment on multiple CUDA powered nodes, is additionally provided in the package. SW#db MPI implementation utilizes MPI nodes by dividing the database into smaller parts so that each node solves only a part of the database. The main node afterwards gathers the results and combines them. Databases are expected to exist on each of the MPI nodes. In this way the data transfer is minimal, since only the end alignments are transferred between the nodes. Except with the mentioned Smith-Waterman algorithm, SW#db library provides alignment using Needleman-Wunsch [14] and semi-global alignment algorithms. Apart from proteins, SW#db can also be used to align against the nucleotide databases. While the existing CUDA accelerated database similarity search software is often focused only on providing alignment scores [5], this library provides both scores and the full alignment paths. SW#db library is intended for external usage in database aligning heuristics, as it provides simple, flexible and powerful API. Dynamic delegation of work and dynamic CPU-GPU communication allows SW#db to significantly lower the algorithm execution time. Another advantage of the API is GPU database preparation in advance for multiple scoring, which in case of multi query database alignment lowers the execution time significantly. Preparation in advance removes data preprocessing and CPU to GPU memory transfer overheads, since it is done only once for multiple queries. Library also provides methods for database alignment with indexes, method for aligning only the selected sequences from the database. These methods can be very useful when used with heuristic database alignment solutions, since almost all of the heuristic solutions rely on sequence alignment algorithms.

## Results

To systematically compare the performance of SW#db with BLASTP, SSW, CUDASW++ (versions 2.0 and 3.1) and SSEARCH, we used a list of proteins of various lengths (**Table 1**) and the ASTRAL dataset [15] as queries and Swiss-prot and Uniref90 as databases. Tests were performed on two configurations: single-GPU (Intel® Core™ i7-4770K CPU, 32 GB RAM, NVIDIA GeForce GTX 780, 256 GB SSD) and multi-GPU (2-socket Intel® Xeon Haswell (E5-2640 v3 @ 2.60GHz) server with 16 cores in total, 128 GB, equipped with 2x NVIDIA K80 GPU cards (4 GPUs in total)). For testing the performances of MPI implementation we used two multi-GPU servers. List of commands and parameters that were run for each program are presented in **S1 Text**.

**Table 1:** The list of Uniprot IDs and lengths of proteins used in performance testing.

Uniprot ID	Length (residues)
O74807	110
P19930	195
B8E1A7	299
Q3ZAI3	390
P18080	513
O84416	607
A9BIH4	727
Q2LR26	804
B4KLY7	980
Q5R7Y0	1465
Q700K0	5124
P0C6V8	6733
P0C6W9	7094
O01761	8081
Q6GGX3	10746
Q9I7U4	18141
Q8WXI7	22152
Q3ASY8	36805

**Fig 3:**
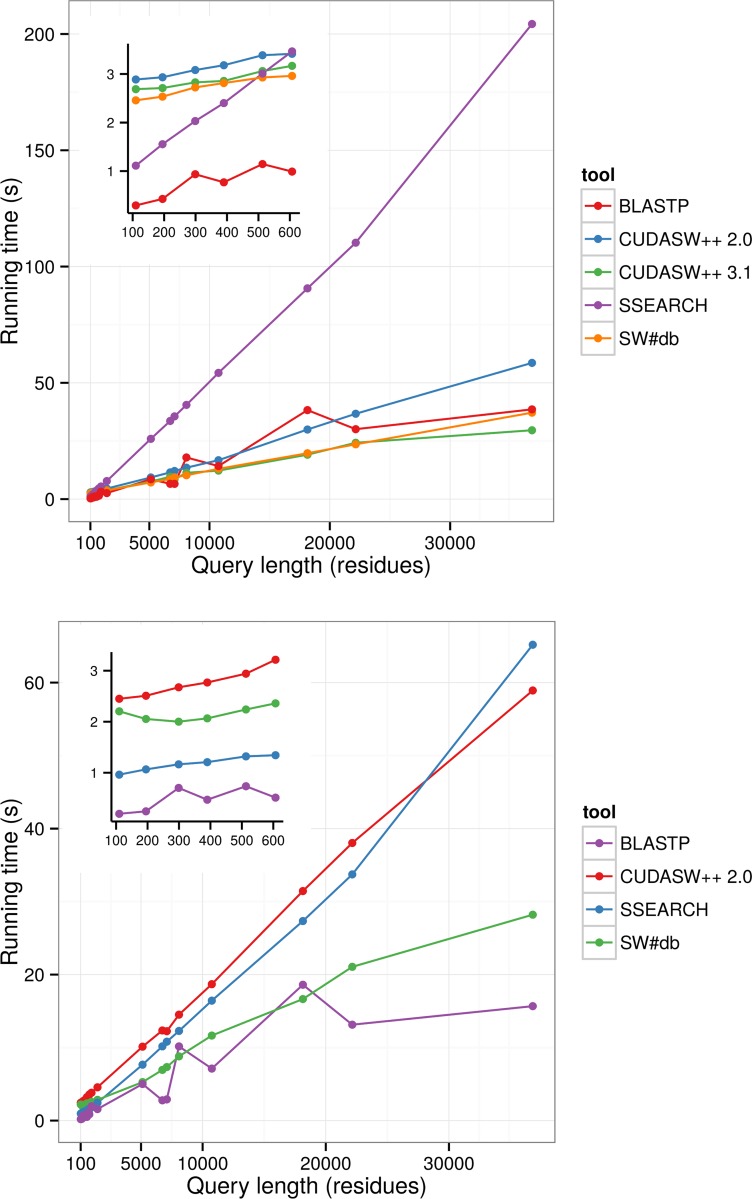
Comparison of SW#db against BLASTP, CUDASW++ v. 2.0, CUDASW++ v. 3.1 and SSEARCH for single-sequence queries of different length on the Swiss-prot database. The insets show detailed results for shorter queries. The upper graph shows results for single-GPU machine (Nvidia GeForce GTX 780). The lower graph shows results for multiple-GPU machine (2 × Tesla K80).

**Fig 4:**
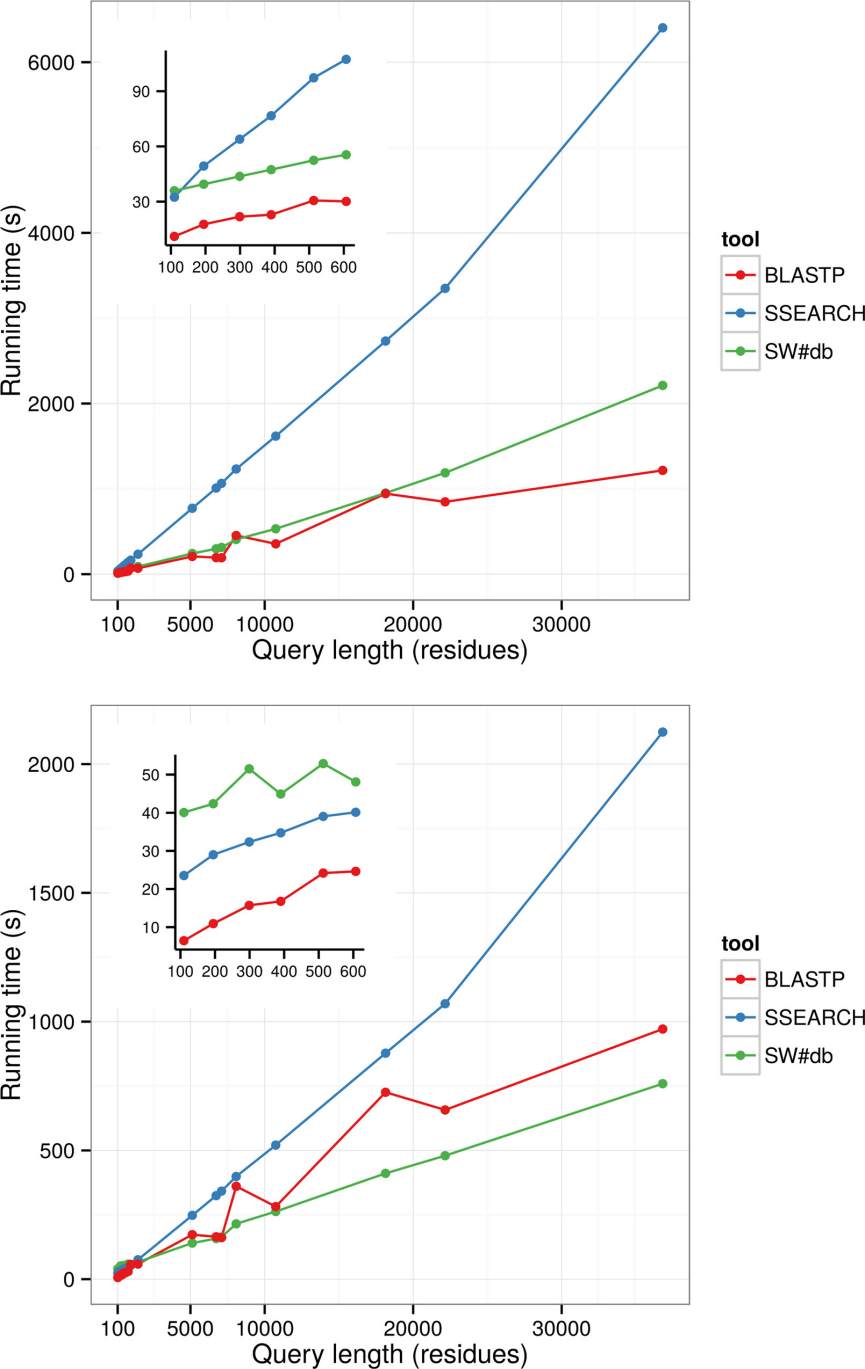
Comparison of SW#db against BLASTP and SSEARCH for queries of different length for UniRef90 database. The insets show detailed results for shorter queries. The upper graph shows results for single-GPU machine (Nvidia GeForce GTX 780). The lower graph shows results for multiple-GPU machine (2 × Nvidia Tesla K80).

Although SW#db is primarily designed to be used for multiple sequence queries, we tested its performances for single queries as well. The results in **Fig 3** and **Fig 4** show that while for shorter queries, up to 600 residues long, CPU based tools BLASTP and SSEARCH are faster, for longer queries GPU based tools are comparable to BLASTP and up to 4 times faster than SSEARCH. The slower running times for shorter queries are expected due to the latency in transferring database to GPU. Additionally, the parallelization for shorter queries is not as efficient as for longer ones. In all tests with the Swiss-prot database SW#db outperforms CUDASW++. For tests with the Uniref90 database we could not run CUDASW++ because this database was too big. We tried running both CUDASW++ v.2.0 and v.3.1. Unfortunately we could not run tests on v.3.1 due to the segmentation fault. We managed to run this version on a configuration with older NVIDIA GTX690 cards and although it running times were similar to the running times of SW#db almost for all protein lengths, except for the lengths longer than 20000 residues where it was slightly faster (**S2 Fig**). **Fig 3** and **Fig 4** do not include results achieved by SSW, because it was much slower than other tools. It was 3 to 15 times slower than the second slowest tool, SSEARCH. We repeated each test 5 times and the presented results are the averages of running times. For each test the standard deviation was below 3%.

The real power of parallelization starts to show for multiple sequence queries. We used all of the above mentioned programs to align the ASTRAL database against the UniprotKB/Swiss-prot and Uniref90 databases. The results are presented in Table 2. It shows that the running times for BLASTP and SW#db are comparable. For the smaller database (Swiss-prot) they are almost equal, while for the longer one (Uniref90) BLASTp is 1.7 times faster. In comparison with other similarity search algorithms based on the Smith-Waterman algorithm, SW#db is faster. It is 4–5 times faster than SSEARCH, 6–25 times faster than CUDASW++ v.2.0 and more than 20 times faster than SSW. In addition we managed to run CUDASW++ v.3.1 on a configuration with older Nvidia GTX 690 cards. CUDASW++ v3.1 was only a slightly faster than version v.2.0 (The results for all tools for this configuration are presented in **S1 Table**).

**Table 2:** Comparison of running times for SW#db, BLASTP, CUDASW++ v2.0, SSW and SSEARCH using ASTRAL database as a query file. We could not run CUDASW++ 3.1 on the both machines (segmentation fault). Both versions of CUDASW++ could not run on Uniref90 due to the size of database. We did not measure running time of SSW for Uniprot90 because it would last too long.

Database	Configuration	Running time (s)				
		SW#db	BLASTP	SSEARCH	CudaSW++ v2.0	SSW
Swiss-prot	Single-GPU; Nvidia GeForce GTX 780 card	3523	3494	15123	23795	87118
Uniref90	Single-GPU; Nvidia GeForce GTX 780 card	123581	73117	490543	-	-
Swiss-prot	Multi-GPU; 2×Nvidia Tesla K80 cards	1264	2210	6063	30174	-
Uniref90	Multi_GPU; 2×Nvidia Tesla K80 cards	41019	29597	164188	-	-

**Fig 5:**
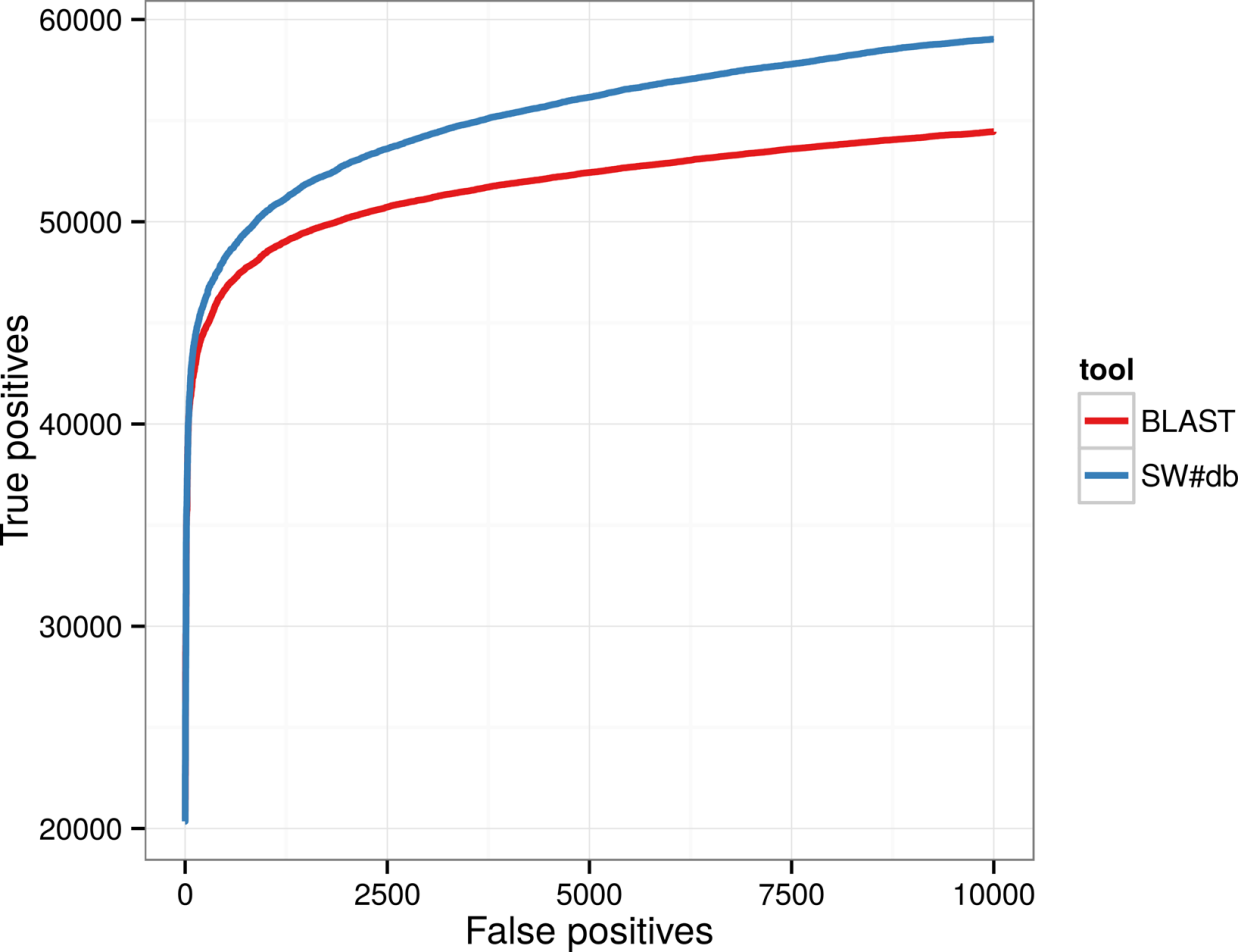
Comparison of sensitivity of BLASTP and SW#db on the Astral/SCOP database.

In addition, we have compared BLASTP with SW#db on the Astral/SCOP compendium database, version 2.04 [16]. For this testing, we created a query set from the subset of Astral sequences. The query set was created by sorting the SCOP domains in a lexicographic order and selecting even numbered sequences as queries. The database consisted of 13042 sequences while the query set contained 6114 sequences. Curves denoting the number of true positives vs. the number of false positives for each algorithm are plotted in Fig 5. The results show that SW#db as an implementation of the Smith-Waterman algorithms is more sensitive than BLAST.

To prove correctness we made the comparison of scores achieved with SW#db and SSEARCH. We randomly selected 100 proteins and aligned them against Swiss-prot database and compared the 10 best scored alignments achieved with these tools. The results in all cases were identical.

## Availability and Future Directions

The source code can be obtained from http://sourceforge.net/projects/swsharp/ and the tool is documented and rigorously tested. We provide both Windows and Linux releases. The minimal recommended architecture is a dual core CPU, over 2.0GHX, 2GB RAM and NVIDIA GPU with Fermi (v2.0) or newer architecture. The further development of SW#db will be focused on the better utilization of parallelization capabilities of both GPU and CPU and on the better load balancing between GPU and CPU.

## Conclusion

In this paper we present the SW#db tool, a parallelised version of exact database search algorithms optimised for multiple queries. Although the emphasis is on the Smith-Waterman algorithm, other exact algorithms such as global and semi-global alignment are provided as well. SW#db is parallelized on both GPU and CPU and it can run on multiple GPUs or on a cluster. The running times for large databases are comparable to the times achieved by BLASTP and at least four times faster than the state-of-the-art parallelized tools used for the same purposes such as SSEARCH, CUDASW++ and SSW. Although it could be used for the protein database search instead of BLASTP when the high sensitivity is required, our main intention was to build a library that could provide fast and exact alignment between queries and a reduced database for various bioinformatics tools.

## Supporting Information

S1 Algorithm Database processing. (DOCX)

S1 Fig Flowchart. (TIFF)

S2 Fig Comparison of SW#DB with BLASTP and SSEARCH for queries of different length and Swiss-prot database. The inset shows detailed results for shorter queries. The results are achieve on a multi-gpu server (Intel® Core(TM) i7-3770 CPU, 16 GB RAM, 2 * NVIDIA GeForce GTX 690, 256 GB SSD).
(TIFF)

S1 Table Comparison of running times for SW#db, BLASTP, CUDASW++ v2.0, CUDASW++ v3.0, SSW and SSEARCH using ASTRAL database as a query file and the Swis-Prot database as target. The results are achieve on a multi-gpu server (Intel® Core(TM) i7-3770 CPU, 16 GB RAM, 2 * NVIDIA GeForce GTX 690, 256 GB SSD).
(DOCX)

S1 Text List of commands and parameters that were run for each program. (DOCX)
