# Supplementary material for: SW#db: GPU-Accelerated Exact Sequence Similarity Database Search
Source: PLoS One. 2015 Dec 31;10(12):e0145857. doi: 10.1371/journal.pone.0145857 (PMC4699916; doi:10.1371/journal.pone.0145857)
Supplement: S1 Algorithm — (DOCX) [file pone.0145857.s001.docx]

**S1 Algorithm. Database processing**

**score(query, database, use_simd)**

short, long = split(database)

exec_in_parallel(CPU_long(), short())

wait_for(short())

stop(CPU_long())

GPU_long(query, long[last_cpu_long ... len(long)], use_simd)

if (use_simd)

database1 = detect_overflows(database)

score(quert, database1, false)

**short()**

exec_in_other_thread(CPU_short())

for (l in 1 ... len(short), step = N

if (l + N > first_cpu_short)

stop(CPU_short())

GPU_short(query, short[l, l + N], use_simd)

**CPU_short()**

for (l in len(short) ... 1)

if use_simd

send_to_thread_pool(CPU_SIMD(query, l))

else

send_to_thread_pool(CPU(query, l))

for (l in len(short) ... 1)

if (should_stop()) stop

wait_for_thread_pool(l)

first_cpu_short = l

**CPU_long()**

for (l in 1 ... len(long))

if use_simd

send_to_thread_pool(CPU_SIMD(query, l))

else

send_to_thread_pool(CPU(query, l))

for (l in 1..len(long))

if (should_stop()) stop

wait_for_thread_pool(l)

last_cpu_long = l
