# Supplementary figures and images for: SW#db: GPU-Accelerated Exact Sequence Similarity Database Search
Source: PLoS One. 2015 Dec 31;10(12):e0145857. doi: 10.1371/journal.pone.0145857 (PMC4699916; doi:10.1371/journal.pone.0145857)

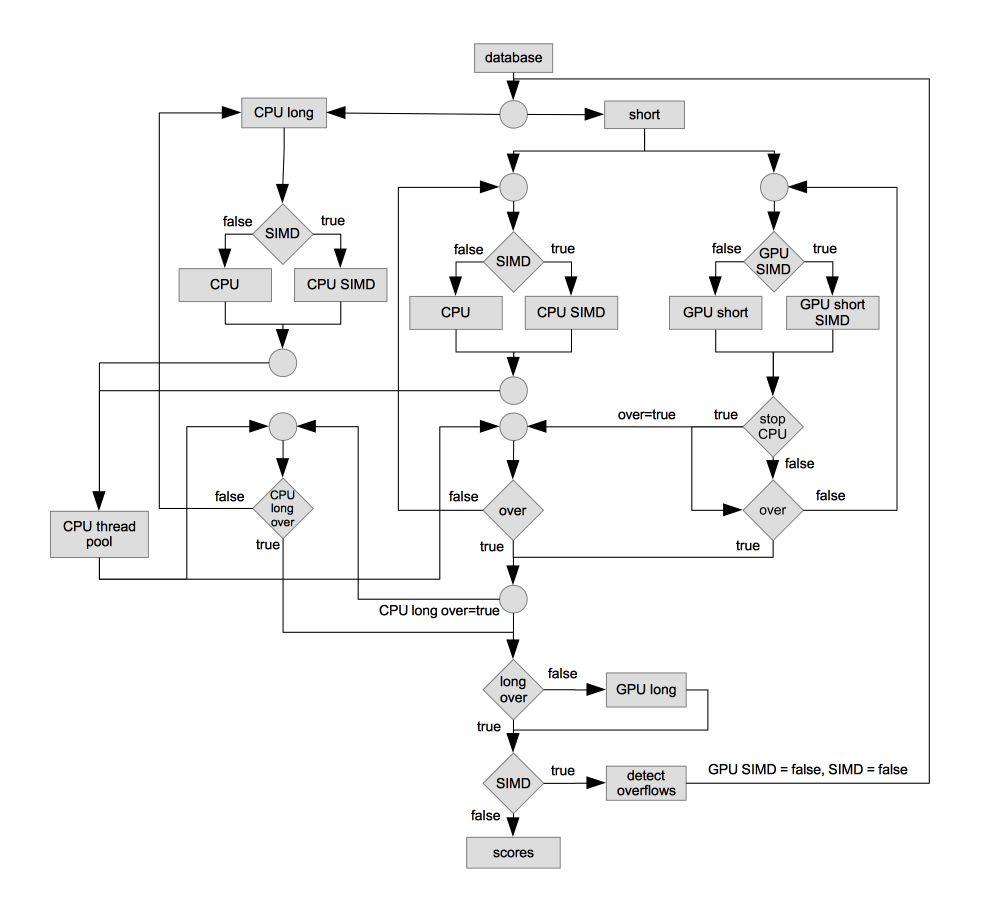

Supplement: S1 Fig — (TIFF) [file pone.0145857.s002.tiff]

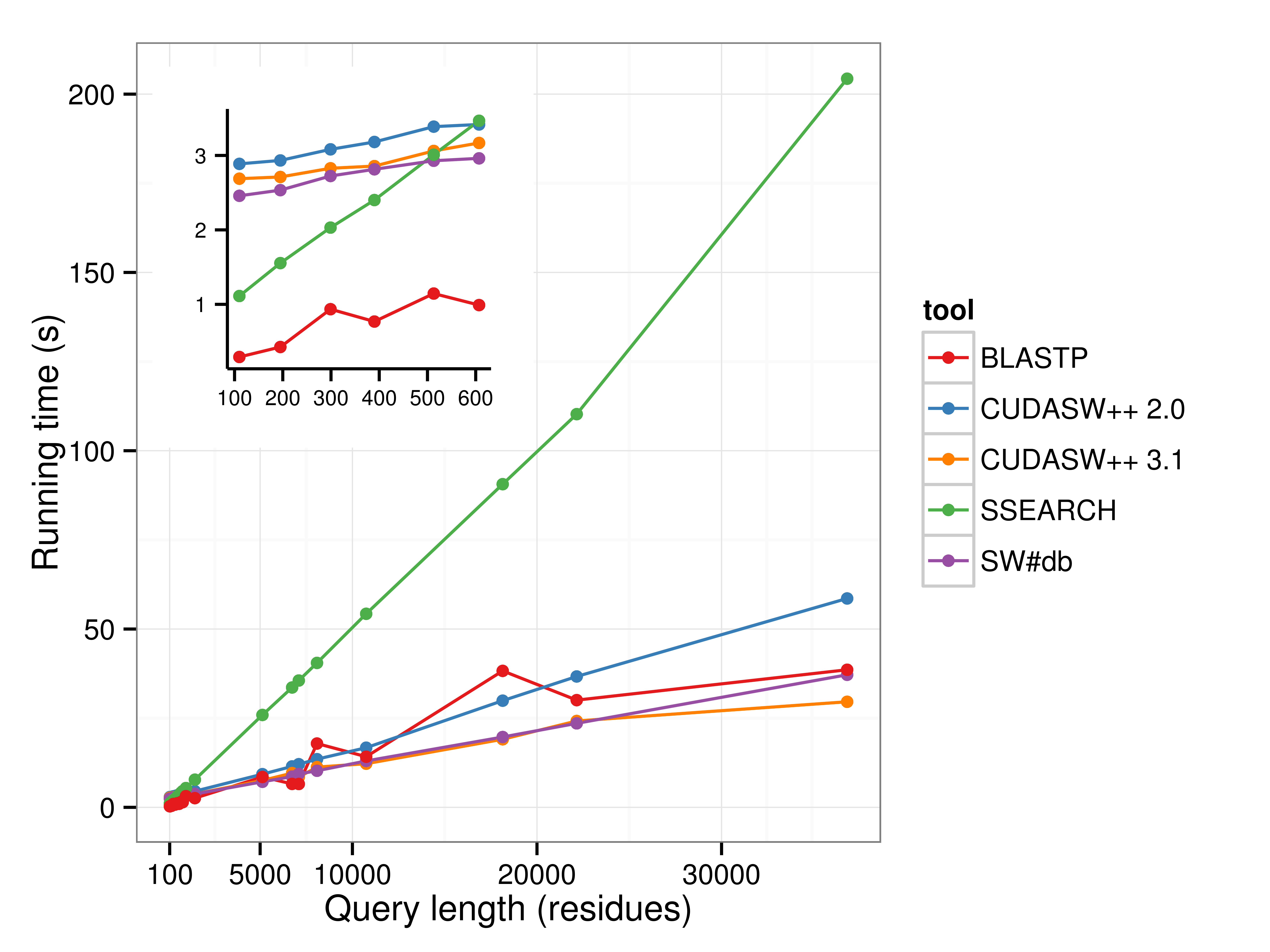

Supplement: S2 Fig — The inset shows detailed results for shorter queries. The results are achieve on a multi-gpu server (Intel® Core(TM) i7-3770 CPU, 16 GB RAM, 2 * NVIDIA GeForce GTX 690, 256 GB SSD). (TIFF) [file pone.0145857.s003.tiff]
