# Supplementary material for: SW#db: GPU-Accelerated Exact Sequence Similarity Database Search
Source: PLoS One. 2015 Dec 31;10(12):e0145857. doi: 10.1371/journal.pone.0145857 (PMC4699916; doi:10.1371/journal.pone.0145857)
Supplement: S1 Table — The results are achieve on a multi-gpu server (Intel® Core(TM) i7-3770 CPU, 16 GB RAM, 2 * NVIDIA GeForce GTX 690, 256 GB SSD). (DOCX) [file pone.0145857.s004.docx]

**S1 Table.** **Comparison of running times for SW#db, BLASTP, CUDASW++ v2.0, CUDASW++ v3.0, SSW and SSEARCH using ASTRAL database as a query file and the Swis-Prot database as target.** The results are achieve on a multi-gpu server (Intel® Core(TM) i7-3770 CPU, 16 GB RAM, 2 * NVIDIA GeForce GTX 690, 256 GB SSD)

| Tools | Running times (s) |
| --- | --- |
| SW#db | 2709 |
| BLASTP | 3018 |
| SSEARCH | 14088 |
| CudaSW++ v2.0 | 32811 |
| CudaSW++ v3.1 | 30545 |
| SSW | 85245 |
