# Supplementary material for: SW#db: GPU-Accelerated Exact Sequence Similarity Database Search
Source: PLoS One. 2015 Dec 31;10(12):e0145857. doi: 10.1371/journal.pone.0145857 (PMC4699916; doi:10.1371/journal.pone.0145857)
Supplement: S1 Text — (DOCX) [file pone.0145857.s005.docx]

**S1 Text. List of commands and parameters that were run for each program.**

SW#db

for f in $(ls data/proteins/* -Sr1); do time ./swsharp/bin/swsharpdb --matrix BLOSUM_62 -g 12 -e 1 -i $f -j dbs/uniref90.fasta > /dev/null 2>&1; done 2> tmp1

SSEARCH

for f in $(ls data/proteins/* -Sr1); do time ./fasta-36.3.6d/bin/ssearch36 -s BL62 -f 11 -g 1 -b 10 -d 0 $f dbs/uniref90.fasta > /dev/null 2>&1; done 2> tmp2

SSW

for f in $(ls data/proteins/* -Sr1); do time ./Complete-Striped-Smith-Waterman-Library/src/ssw_test -a b62 -p -o 12 -e 1 $f dbs/uniref90.fasta > /dev/null 2>&1; done 2> tmp3

BLASTP

for f in $(ls data/proteins/* -Sr1); do time ./ncbi-blast-2.2.27+/bin/blastp -gapopen 12 -gapextend 1 -matrix BLOSUM62 -num_alignments 10 -num_threads 8 -query $f -db dbs/uniref90.fasta > /dev/null 2>&1; done 2> tmp4

CUDASW++

for f in $(ls data/proteins/* -Sr1); do time ../cudasw++v2.0.10/cudasw -mat blosum62 -gapo 11 -gape 1 -topscore_num 10 $f dbs/uniref90.fasta > /dev/null 2>&1; done 2> tmp5
